# Cardiovascular risk factor assessment in late‐onset seizures: A study protocol to assess the value of structured intervention

**DOI:** 10.1002/epi4.12987

**Published:** 2024-06-14

**Authors:** David Larsson, Signild Åsberg, Johan Sundström, Petrea Frid, Johan Zelano

**Affiliations:** ^1^ Department of Neuroscience and Physiology, Sahlgrenska Academy University of Gothenburg Gothenburg Sweden; ^2^ Department of Neurology Sahlgrenska University Hospital, Member of Epicare Gothenburg Sweden; ^3^ Wallenberg Center for Molecular and Translational Medicine University of Gothenburg Gothenburg Sweden; ^4^ Department of Medical Sciences Uppsala University Uppsala Sweden; ^5^ The George Institute for Global Health, University of New South Wales Sydney New South Wales Australia; ^6^ Department of Neuroscience University of Lund Lund Sweden

**Keywords:** comorbidity, late‐onset epilepsy, stroke

## Abstract

**Objective:**

A growing body of evidence suggests patients with late‐onset seizures are at an increased risk of stroke, but the potential for reducing cardiovascular morbidity through risk factor screening and management is unknown. We aim to determine whether individuals with new‐onset unprovoked seizures after middle age should undergo vascular risk assessment. The long follow‐up needed to assess stroke risk and the known benefit of vascular risk factor modification make a standard RCT logistically and ethically challenging. Instead, we propose and have developed a protocol for a cluster project assessing the effect of vascular risk factor screening in an intervention trial as well as a cohort study.

**Methods:**

Participating neurology clinics will implement standard cardiovascular risk factor assessment into the routine evaluation for individuals aged ≥50 years attending their first specialized consultation after an unprovoked seizure, excluding those with progressive brain disease. The project has two interlinked components: a prospective single group trial, in which risk factor assessment is performed and subsequent management is followed for one year; and a register‐based cohort study examining the long‐term effects of the intervention on a system level by comparing patients attending initial consultations in the 2 years after start of the study, with patients seen in the four preceding years at the same clinics.

**Analysis:**

The primary outcome of the intervention trial is the proportion of patients receiving subsequent pharmacological treatment. The primary outcome of the cohort study is the incidence of acute stroke in the Swedish Stroke Register.

**Ethics and Dissemination:**

Swedish Ethical Review Authority approval (which is valid for 2 years only) will be sought when funding is obtained. The results will be disseminated through peer‐reviewed scientific publications.

**Registration Details:**

The study will be registered at clinicaltrials.gov.

**Plain Language Summary:**

A first seizure in a middle‐aged or older person indicates a higher risk of stroke. It is not known whether investigating and treating blood pressure, blood cholesterol, or similar risk factors after a first seizure is an effective way to prevent stroke. A traditional clinical study would need too many patients and it would be unethical not to treat the control group. We have designed a study in which participating neurology departments change their practice to test and treat vascular risk factors. Patients are then compared to historic controls using registered data.

**Key points**

- The proposed study investigates whether standardized vascular risk factor assessment is warranted in patients with late‐onset seizures.
- The study has a short‐term analysis of proportion receiving therapy after the intervention.
- The study has a long‐term analysis of stroke risk compared to historic controls.
- Swedish comprehensive registers are used for the long‐term outcome, minimizing loss to follow‐up.
- A limitation is the non‐randomized design, but untreated controls are unethical given the benefits of vascular risk factor modification.

## 1 INTRODUCTION

### 1.1 Background

Cerebrovascular disease is the most common cause of epilepsy after middle age. Interestingly, seizures can also precede stroke.^1^, ^2^, ^3^, ^4^, ^5^ UK primary care data indicated a nearly threefold increased risk of stroke after a first seizure in those aged ≥60 years,^4^ a finding that was recently replicated in Swedish population wide register data, albeit with a slightly lower risk (HR 2.27, 95%CI: 1.87–2.75).^1^ The relative increase in risk was greater for intracerebral hemorrhage (HR 2.51, 95%CI: 2.08–3.04) than ischemic stroke (HR 1.68, 95%CI: 1.56–1.80).^1^ Vascular risk factors increase the risk of late‐onset epilepsy,^6^ so a plausible explanation is that late‐onset seizures reflect occult cerebrovascular disease that has not yet resulted in a clinical stroke. This notion is further supported by a higher prevalence of silent cerebrovascular disease on imaging in patients with late‐onset seizures than in healthy controls.^7^ The highest increase in stroke risk is seen in the first year following the first seizure.^1^ Another contributing factor could be detrimental effects of antiseizure medications (ASM) on vascular risk prevention, like interactions with lipid‐lowering drugs.^8^

Given the emerging evidence of increased stroke risk after late‐onset seizures, the potential benefits of vascular risk factor management in such patients are being discussed,^7^, ^9^ but evidence on the optimal nature and effect of these interventions is lacking. In addition to stroke, other cardiac events like arrythmias or sudden cardiac death are associated with epilepsy or ictogenic mechanisms^10^, ^11^ but whether vascular risk factor intervention mitigates such risks is unknown. Current clinical practice does not consider vascular risk factors in management of first seizures. It is simply not known whether detection and treatment of hypertension and/or optimization of serum lipids is beneficial or not in this patient group.

### 1.2 Rationale

Since there can be a long latency between a first seizure and stroke, and since benefits of treatment of many vascular risk factors are well established, a standard RCT is logistically and ethically challenging. Swedish comprehensive health registers allow a multicenter register‐based randomized controlled trial (RRCT) design, but even with the advantages of that approach recruitment would be challenging. A 1:1 randomized study that assumes a 10‐year cumulative stroke rate of 10% and a 30% relative risk reduction from the intervention would require 247 events and 4494 participants to be completed in 5 years (*α* = 0.05; *β* = 0.2; censoring rate = 0.1). Since all potentially recruitable cases in Sweden are roughly 2000 candidates per year (incidence of late‐onset seizures: ~60 per 100 000), this is not a feasible study design. Furthermore, the benefits of treatment of hypertension and diabetes as well as smoking cessation are well known, making it ethically dubious to withhold such interventions from a control group. Since the research question is not whether vascular risk factor assessment is beneficial, but whether vascular risk assessment specifically in the work‐up of late‐onset seizures is beneficial on a system level, we decided on a cluster‐based design.

### 1.3 Objectives

This project aims to assess the impact of implementing vascular risk factor assessment (screening) in the work‐up of individuals with a new‐onset unprovoked seizure after age 50. The vascular risk assessment equals the opportunistic screening that can already be done during health visits according to Swedish guidelines. Any vascular risk factor modification after the assessment is done according to existing guidelines. We aim to assess both the proportion of patients that receive pharmacotherapy as a result of the assessment and the effect on long‐term stroke risk.

## 2 METHODS AND ANALYSIS

### 2.1 Study design

**FIGURE 1:**
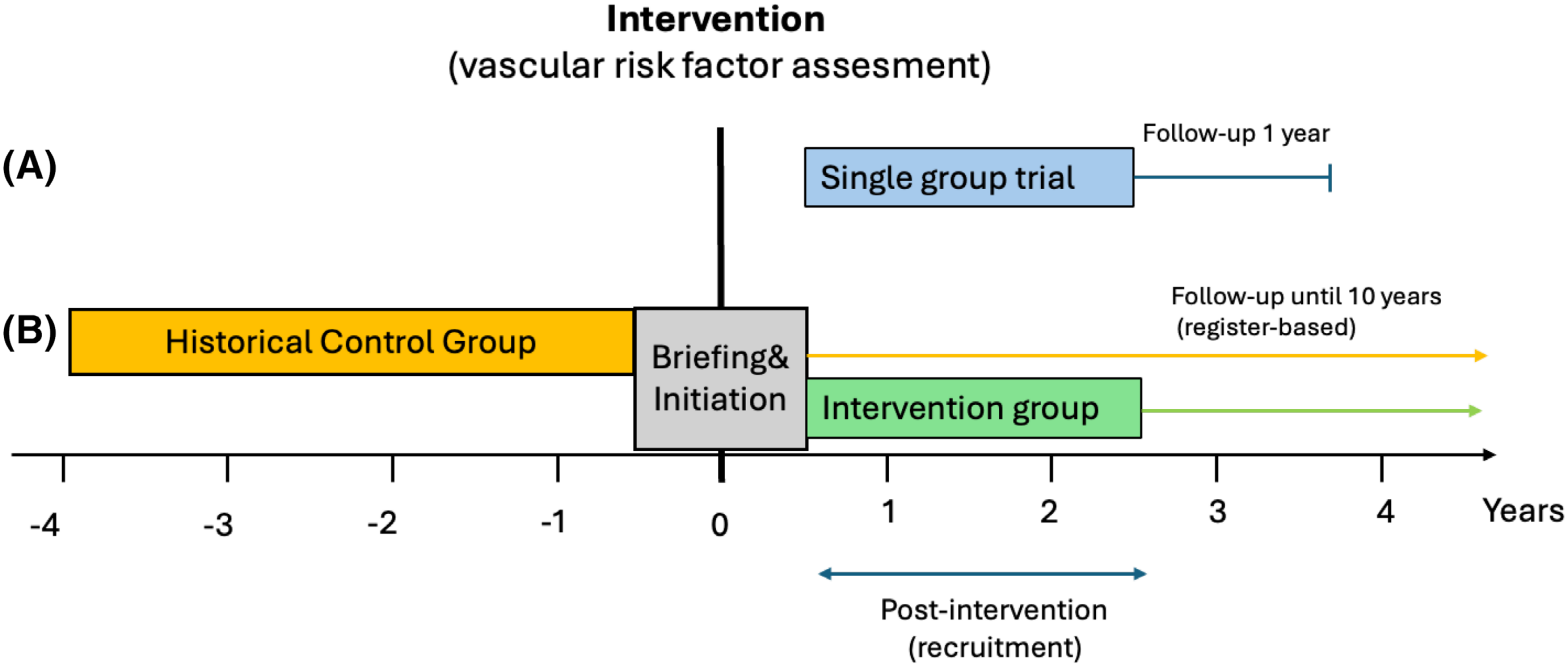
The study design comprises two main components: (A) a prospective single group trial with a one‐year follow‐up assessing the prevalence of vascular risk factors and the impact of screening on subsequent management and (B) a register‐based cohort study examining the long‐term effect on vascular risk by comparing patients seen after introduction of the new standard to historical controls from the same sites.

Participating neurology clinics in Sweden will introduce cardiovascular work‐up as the standard of care following late‐onset seizures. Subsequently, we will use pseudonymized data from comprehensive national registers to compare outcome differences between new patients (those attending first visits) before and after the practice change. In addition, following the new standard of care implementation, all eligible patients are offered to participate in a one‐year prospective trial that examines the prevalence of modifiable vascular risk factors and subsequent cardiovascular treatment (Figure 1).

### 2.2 Intervention: Evaluating modifiable vascular risk factors

The evaluation of vascular risk factors aims to identify and manage modifiable vascular risk factors early, in line with the current ESC guidelines on cardiovascular disease prevention.^12^ Like most work‐up protocols, investigators may refrain from evaluating selected cases when such intervention is deemed inappropriate or the benefit of potential cardiovascular prevention is minimal. To promote adherence to the practice change, relevant health care staff will receive information during a 6‐month “briefing and implementation phase,” during which they can also report barriers to adherence and suggest site‐specific logistical improvements.

The examinations included in the work‐up are at the investigator's discretion to align with existing local guidelines; however, the minimum requirements are listed in Table 1. A registered nurse or a nursing associate will generally be available with each initial consultation to conduct any ordered examinations, minimizing the need for additional visits. Using composite risk assessment tools, for example, SCORE2/SCORE2‐OP, is advised to guide preventive measures in individuals without already established cardiovascular disease. Investigators are free to give lifestyle advice and prescribe medications according to local or international guidelines and to tailor treatment according to the patient's preferences; the study protocol does not specify any management strategies for identified conditions beyond what existing Swedish guidelines already recommend. Participating sites will have regular education on what the guidelines stipulate regarding blood pressure targets, lipid level targets, and smoking cessation.

**TABLE 1:** Mandatory components of the vascular risk factor evaluation.

Procedure
Family history of vascular disease
Tests/examinations (order or obtain from medical records [past 3 months])
Weight
Height
Waist circumference
12‐lead ECG
Resting and standing blood pressure
Total cholesterol
HDL cholesterol
LDL cholesterol
HbA1c
High sensitivity CRP
Serum creatinine
Urine albumin‐to‐creatinine ratio
Medical history
Atherosclerotic cardiovascular disease
Atrial fibrillation
Congestive heart failure
Diabetes mellitus
Hypertension
Kidney disease
Life‐style risk factors (smoking, exercise)

### 2.3 Sample selection

Patients undergoing their first‐ever specialized assessment for unprovoked seizures or epilepsy with onset after age 50, who also receive a vascular risk factor evaluation (minimum requirements are outlined in Table 1) in conjunction with their initial consultation (within 1 month), are invited to participate in a prospective one‐year trial. Individuals unable to provide informed consent and those with established progressive brain disease, that is, brain tumor or neurocognitive disorder, will be excluded. Notably, we will not exclude patients already on primary or secondary cardiovascular prevention; thus, the study will include groups for whom the benefit from the intervention may be limited.

In addition, we will conduct a system‐level register‐based cohort study based on data from the National Patient Register (NPR), which covers information on all specialized health care encounters in Sweden, and the Swedish Prescribed Drug Register (SPDR), which encompasses details of all prescribed drugs dispensed at pharmacies. Patients aged ≥50 with a first‐ever outpatient appointment to one of the participating clinics and a first‐listed diagnostic code for seizures or epilepsy (ICD‐10: R568, G40) are included. Exclusion criteria will be (i) preexisting seizures/epilepsy, as demonstrated by a registered diagnostic code for seizures (ICD‐10: R568, G40, G41) or a dispensed antiseizure medication (ATC‐code: N03) more than 3 months before the initial consultation, (ii) a registered diagnostic code for stroke or TIA (ICD‐10: I61, I63, I64, or G45 except G454) before the initial consultation, or (iii) progressive brain disease, as indicated by diagnostic codes or drug prescriptions suggestive of brain tumors (ICD‐10: C71, D33, D43, C793) or neurocognitive disorders (ICD‐10: F00‐F03, F051, G30, G318, G912; ATC code: N06D). The only difference in eligibility criteria between the intervention group and the control group will be the period of recruitment; the former will consist of patients with their initial consultation in the 2 years following the “briefing and implementation phase” of the center, whereas the control group will encompass those seen in the four preceding years at the same site.

### 2.4 Outcomes

The purpose of the prospective single group trial is to assess the prevalence of vascular risk factors and the impact of screening on subsequent management and vascular risk. The primary outcome is the proportion of cases receiving pharmacological intervention following the vascular risk factor evaluation; pharmacological intervention is defined as the initiation or dose adjustment of antihypertensive agents, antidiabetic agents (including SGLT2 inhibitors used for other indications), lipid‐lowering agents, antiplatelet agents, or anticoagulants (Table 2). Secondary outcomes are as follows: (i) proportion of cases where the screening procedure uncovers previously undetected vascular risk factors, subdivided into hypertension, diabetes mellitus, moderate‐to‐severe chronic kidney disease, and atrial fibrillation, (ii) alterations in modifiable vascular risk factors at the one‐year follow‐up compared to baseline measurements, (iii) changes in SCORE2/SCORE2‐OP risk scores at the one‐year follow‐up, specifically in individuals without established cardiovascular disease, (iv) proportion of participants with adverse effects following pharmacological intervention, (v) proportion with arrythmias or QT‐prolongation captured during admissions, and (vi) proportion of participants remaining on initiated treatment at the 12‐month follow‐up.

**TABLE 2:** Variables and outcomes for the two analyses.

Variables	Primary outcome	Secondary outcomes
One‐year single group prospective analysis		
See Table 1	% with pharmacological intervention	Hypertension, diabetes mellitus, moderate‐to‐severe chronic kidney disease, atrial fibrillation, alterations in modifiable vascular risk factors compared to baseline, changes in SCORE2/SCORE2‐OP at the one‐year follow‐up, % adverse effects
Matched cohort long‐term analysis		
Demographics, vascular disease, comorbidities that can cause seizures, medical treatments	Stroke	Major adverse cardiovascular events, all‐cause mortality, stroke severity (NIHSS at stroke admission; modified Rankin Scale 3 months post‐stroke), conduction block/arrhythmia, and occurrence of recurrent seizures

The cohort study focuses on long‐term risk of vascular events, at least 5 years. The primary outcome is a registered acute stroke, measured with yearly extractions from the Swedish national stroke register (95% coverage of acute strokes in Sweden). We will also measure several secondary outcomes, including major adverse cardiovascular events, a composite measure of the variables stroke (primary outcome), myocardial infarction (ICD‐10 I21–I22 in NPR or death from myocardial infarction in the CDR), and cardiovascular death (an ICD‐10 code of I0‐I99 [diseases of the circulatory system] as the underlying cause of death). Other secondary outcomes include all‐cause mortality, stroke severity (NIHSS at stroke admission; modified Rankin Scale 3 months post‐stroke), arrythmias or conduction disorder (relevant ICD‐10/procedure codes suggestive of conduction disorder, arrhythmia or cardiac arrest^13^), and occurrence of recurrent seizures, captured by the NPR (hospital admissions [inpatient care] or emergency room visits with a seizure‐related diagnostic code [ICD‐10: R56.8, G40, or G41] as the first‐listed diagnosis). Given the interaction potential and adverse effects of several ASMs on vascular risk, we will also track ASM treatment by prescription data, as previously described.^14^

### 2.5 Data analysis plan

Descriptive statistics will be presented as median (interquartile range) or number (%). For the single group study, we will use the normal approximation method to calculate 95% confidence intervals for proportions, including the primary outcome. Comparisons between baseline and one‐year follow‐up measurements will involve paired analyses, for example, McNemar's test for categorical data and paired t‐test or a non‐parametric equivalent for continuous data, depending on the data distribution. The analyses will be stratified by age groups, sex, and previously established cardiovascular risk factors. All tests will be two‐sided and considered statistically significant at *p* < 0.05.

The cohort study examines time to the different events of interest. We will use the Kaplan–Meier method and Cox proportional hazards regression to evaluate all‐cause mortality. For other events of interest, death will be accounted for as a competing risk. Survival times are calculated from the initial consultation until either the event of interest, death or study end, whichever comes first. To compare baseline group differences, we will utilize the Fisher exact test, the *χ* ^2^ test, or the Mann–Whitney *U* test, depending on the data characteristics. We do not anticipate any significant group differences; however, we will perform univariable and multivariable regression analyses to identify and adjust for any relevant covariates (age, sex, medical history, prescriptions of drugs used for cardiovascular prevention). Subgroup analyses will comprise previously “healthy” individuals (no drug prescriptions related to cardiovascular prevention) and individuals without established cardiovascular disease (defined as not being prescribed antiplatelet agents or anticoagulants). Analyses will also be stratified by age groups and sex.

### 2.6 Sample size calculation

The incidence of late‐onset seizures is about 60 per 100 000 person‐years.^15^ Among these cases, approximately two‐thirds do not have an identifiable cause, such as a brain tumor. Participating health service regions cover about 5 million inhabitants and should have at least 1000 potential cases yearly. For the primary outcome of the single group analysis (proportion of cases undergoing pharmacological intervention), we aim for a margin of error less than 5%, which would translate to a minimum sample size of 385 (using an estimated proportion of 50%; for lower proportions, the required sample size generally decreases). For the cohort study, a 10‐year stroke risk of 10% and an effect of the intervention of 30% risk reduction translate to a need for 1021 participants post‐intervention and 2045 participants pre‐intervention to detect a difference in 10 years. Participating sites should cover approximately 1000 cases in the right age group per year, allowing inclusion of up to 2000 patients over 2 years and 4000 historic controls over 4 years. The vascular risk is not evenly distributed over time, the risk is greatest in the first years, but we should be able to detect a difference after 5–10 years.

### 2.7 Patient and public involvement

The initiation phase will include contact with patient organizations in epilepsy and stroke in each participating health care region, to inform them about the study. Patient organizations will also be contacted for dissemination of the results after the study.

## 3 ETHICS AND DISSEMINATION

The primary ethical consideration is that the screening process and the potential risk factors that may be identified could raise concern among the patients, but vascular risk factor screening is generally not perceived as stressful.^12^ This concern must be weighed against the possibility of preventing morbidity and mortality in patients with late‐onset seizures. Regarding safety considerations, opportunistic vascular risk factor assessment at health contacts is already advised in Sweden, the components of the intervention are already in clinical use, and all relevant drugs that may be prescribed are approved. Generally, side effects are mild and well‐documented. Importantly, the study protocol does not advocate any particular risk factor management strategies beyond what existing guidelines already recommend. The final study protocol, including the final versions of the informed consent form and other materials provided to participants, must be approved by the Swedish Ethical Review Authority. Any subsequent substantial changes to the protocol must be approved by all responsible investigators and the Swedish Ethical Review Authority before they can be implemented. Since ethical approval in Sweden requires resources and is only valid for 2 years, we will submit the ethical application only once funding has been obtained. We confirm that we have read the Journal's position on issues involved in ethical publication and affirm that this report is consistent with those guidelines.

### 3.1 Consent

Informed consent will be mandatory for participation in the single group trial. The principal investigator at each site will ensure that subjects receive complete oral and written information about the study, its purpose, risks and benefits, and inclusion and exclusion criteria. The subject and the investigator shall then both sign the informed consent form (electronic signing will be available). Participants are free to discontinue participation at any time without providing a reason. The analysis of the long‐term stroke risks in the cohort study is an observational study using pseudonymized register data. For studies utilizing pseudonymized register data, the Swedish Ethical Review Authority typically grants a waiver of the informed consent requirement.

### 3.2 Data access and confidentiality

Data collection for the single group study involves Electronic Case Report Forms (e‐CRFs) and recording relevant information in medical records. An electronic data capture system, REDCap, will be used for data management. The study database will be held at Sahlgrenska University Hospital, Gothenburg, Sweden. All data are stored in a pseudonymized manner. The subject information and the informed consent form will explain how study data are stored to maintain confidentiality in accordance with the General Data Protection Regulation (GDPR) and other relevant national data legislation. The National Board of Health and Welfare will provide pseudonymized register data for the cohort study.

### 3.3 Dissemination plan

The results will be disseminated through peer‐reviewed scientific publications and presentations at relevant conferences.

## AUTHOR CONTRIBUTIONS

SÅ, JS, PF, and JZ have been on the planning committee and contributed to the study design and protocol drafting. DL has contributed to study design, protocol, and drafting of the manuscript. JZ has been responsible for conceptualization, design, protocol, and manuscript preparation.

## FUNDING INFORMATION

This work was supported by the Swedish Research Council planning grant number 2020‐06198.

## CONFLICT OF INTEREST STATEMENT

DL declares no conflicts of interest. PF declares no conflicts of interest. SÅ has received institutional research grants and lecture fees to her institution (AstraZeneca, Boehringer‐Ingelheim, Bristol Myers Squibb, Institut Produits Synthese), outside the current project. JS is a shareholder of Symptoms Europe AB and Anagram kommunikation AB, outside the current project. JZ has received speaker honoraria from UCB and Eisai and has as an employee of Sahlgrenska University Hospital (no personal compensation) been investigator in clinical trials sponsored by Bial, UCB, GW Pharma, SK life science, and Angelini Pharma. We confirm that we have read the Journal’s position on issues involved in ethical publication and affirm that this report is consistent with those guidelines.

## Data Availability

Being a study protocol, there is no data.
